# Reduced nephrin tyrosine phosphorylation impairs podocyte force transmission and accelerates detachment in disease

**DOI:** 10.1016/j.isci.2025.112673

**Published:** 2025-05-14

**Authors:** Casey R. Williamson, Claire E. Martin, J. Dinesh Kumar, Peihua Lu, Laura A. New, Alice Y. Wang, Nils M. Kronenberg, Malte C. Gather, Paul A. Reynolds, Nina Jones

**Affiliations:** 1 Department of Molecular and Cellular Biology, University of Guelph, Guelph, ON N1G 2W1, Canada; 2 SUPA, School of Physics and Astronomy, University of St. Andrews, KY16 9SS St. Andrews, UK; 3 Biomedical Sciences Research Complex, University of St. Andrews, KY16 9ST St. Andrews, UK; 4 Department of Chemistry, Humboldt Centre for Nano- and Biophotonics, University of Cologne, 50939 Cologne, Germany; 5 Cologne Excellence Cluster on Cellular Stress Responses in Aging-Associated Disease (CECAD), University of Cologne, 50931 Cologne, Germany; 6 School of Medicine, University of St. Andrews, KY16 9TF St. Andrews, UK

**Keywords:** Biochemistry, Cell biology, Specialized functions of cells, Biomechanics

## Abstract

Podocytes are specialized kidney cells that form the slit diaphragm (SD), an intercellular filtration barrier against plasma protein loss. The SD is subject to significant mechanical strain which can be amplified in disease, leading to podocyte detachment. One key SD protein that might intercept mechanical strain and transmit adhesion signals is nephrin, although its influence on podocyte force transmission remains uncharacterized. Using immunoblotting and elastic resonator interference stress microscopy (ERISM), we demonstrate that nephrin clustering induces rapid podocyte force transmission and adhesion protein activation (paxillin, FAK, and p130Cas), which require nephrin tyrosine phosphorylation at its three YDxV motifs. Furthermore, using a model of diabetic nephropathy to amplify mechanical stress *in vivo*, we show that abolishing phosphorylation at YDxV tyrosines leads to exacerbated proteinuria, glomerular hypertrophy, and podocyte detachment. Altogether, this study links nephrin phosphorylation with force transmission, which is likely critical to maintaining podocyte adhesion during many renal pathologies.

## Highlights

• Nephrin controls podocyte force transmission via phosphorylation of its YDxV motifs
• Nephrin phosphorylation at YDxV motifs leads to focal adhesion protein activation
• Loss of YDxV phosphorylation *in vivo* exacerbates hallmarks of diabetic nephropathy
• Diabetic injury without YDxV phosphorylation leads to increased podocyte detachment

## Graphical abstract

**Figure:**
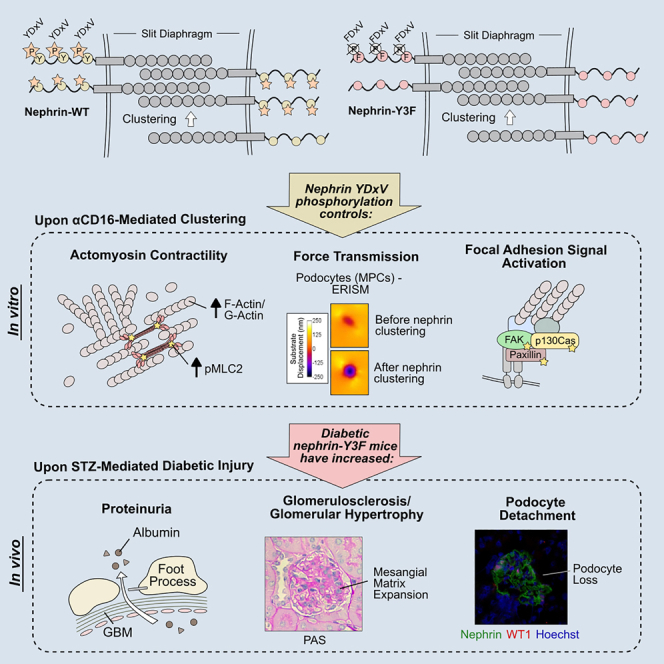

## Introduction

Podocytes are highly specialized kidney cells that function as an essential component of the glomerular filtration barrier (GFB). Located on the outer surface of the capillary wall, podocytes must strongly adhere to the underlying glomerular basement membrane (GBM) to counter hemodynamic stress forces and uphold filtration.^1^^,^^2^ To maintain these functions, podocytes extend interdigitating, actin-rich foot processes (FPs) that are bridged by a modified adherens junction known as the slit diaphragm (SD).^1^^,^^3^ The SD operates as a filter against plasma proteins, while also acting as a major signaling hub of actin organization.^4^^,^^5^^,^^6^^,^^7^ Increased mechanical strain from various kidney injuries can negatively alter SD architecture, leading to FP effacement, GBM thickening, podocyte detachment, and proteinuria.^8^^,^^9^^,^^10^ Because podocytes are terminally differentiated with a limited ability for regeneration,^10^ clinical treatments become increasingly difficult as podocytes are progressively lost. Thus, there is great interest in elucidating the mechanisms by which podocytes maintain adhesion during kidney health and disease.

To withstand the intense biomechanical environment of the glomerulus, podocytes must generate contractile forces and cytoskeletal signaling responses that promote adhesion.^11^ Podocytes rely on focal adhesions (FAs) as key centers of cell-matrix adhesion, which are largely coordinated by activated integrin heterodimers.^12^ Here, paxillin binds β-integrins and works synergistically with the mechanosensors FAK and p130Cas to regulate actin reorganization and actomyosin contractility.^12^^,^^13^^,^^14^^,^^15^^,^^16^ A primary driver of contractile force transmission in podocytes is non-muscle myosin IIa (NM-IIa),^17^^,^^18^ controlled by the phosphorylation of myosin light chain 2 (MLC2).^19^ Collectively, dynamic force transmission is linked with FA maturation and turnover to ensure the integrity of podocyte attachment to the GBM.^12^ At present however, the nature of FA signaling *in vivo* remains unclear, and it is largely unknown if force transmission and adhesion activation can also be stimulated by the SD.

One key SD protein that likely transmits mechanical responses is the nephrin transmembrane receptor. With its extracellular region positioned across the filtration slit, nephrin is directly exposed to flow-induced shear stress, as well as tensile stress from capillary dilation. Moreover, within its cytoplasmic region, nephrin undergoes tyrosine phosphorylation on three YDxV motifs that are crucial for actin remodeling^20^^,^^21^ and recovery from podocyte injury.^22^^,^^23^^,^^24^^,^^25^ Clustering of nephrin molecules at the SD, either through proximity and/or *trans*-interaction with nephrin on opposing FPs, triggers phosphorylation (specifically on Y1176, Y1193, and Y1217 in humans).^20^ These phosphorylated motifs have particular affinity for Nck adaptors,^20^^,^^26^ which have been shown to induce cortical actomyosin contractility and actin polymerization through formation of phase-separated clusters with N-WASP and Arp2/3.^27^ Several downstream effectors of Nck are also implicated in FA dynamics, notably FAK, p130Cas, α-actinin-4, and Arp3.^22^^,^^23^^,^^24^^,^^25^ Nonetheless, a link between nephrin signaling and force transmission and/or FA crosstalk in podocytes has yet to be fully elucidated.

Here, we demonstrate that nephrin clustering initiates a signaling cascade that activates actomyosin contractility and FA changes that are dependent on the YDxV tyrosines. Furthermore, using continuous force monitoring microscopy, we show that nephrin clustering in podocytes induces rapid substrate displacement, and this effect is not seen with a mutant lacking these tyrosines. Lastly, using an *in vivo* model of diabetic nephropathy to mimic pathological mechanical strain, we show that YDxV tyrosine phosphorylation is required to prevent podocyte detachment from the GBM and mitigate proteinuria. Collectively, these findings demonstrate that nephrin signaling induces dynamic changes in podocyte force transmission, and loss of this adaptive phosphorylation leads to podocyte detachment and advanced kidney injury.

## Results

### Nephrin phosphorylation regulates actomyosin contractility

**Figure 1:**
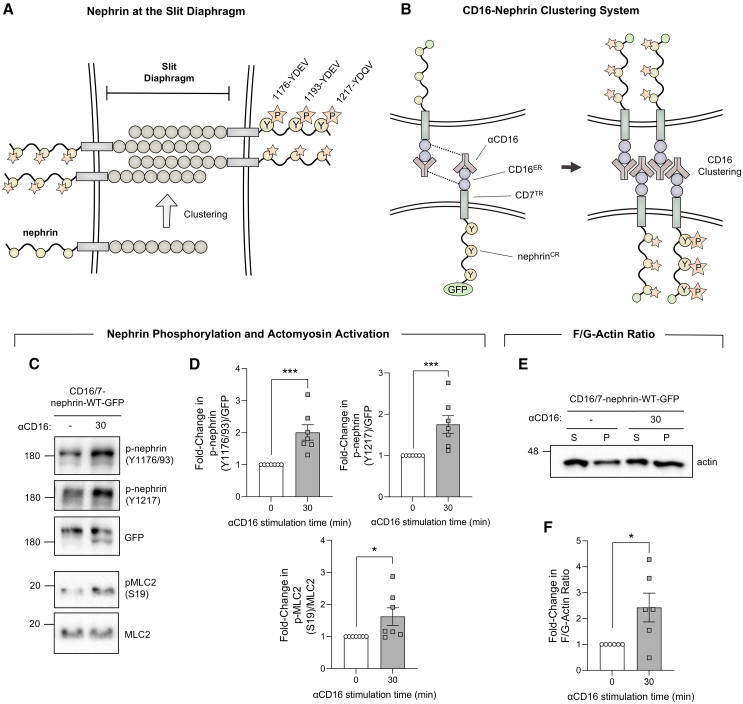
Nephrin clustering in podocytes increases actomyosin contractility and actin polymerization (A) Nephrin clustering at the slit diaphragm leads to its phosphorylation at Y1176, Y1193, and Y1217 (human residue numbers) within YDxV motifs. (B) The CD16 clustering system utilizes a chimeric protein of the CD16 extracellular region (ER), CD7 transmembrane region (TR) and a GFP-tagged nephrin cytoplasmic region (CR). Clustering of nephrin via an anti-CD16 antibody (αCD16) then induces nephrin tyrosine phosphorylation. (C) Western blot panel showing nephrin and myosin light chain 2 (MLC2) phosphorylation following nephrin clustering in mouse podocyte cells (MPCs). (D) Densitometry of western blots shown in (C), normalized to GFP or total MLC2 (*n* = 7). (E) Western blot following actin pelleting assay in MPCs, where a greater proportion of filamentous actin (F-actin) is detected in the pellet (P) after nephrin clustering. Globular actin (G-actin) is found within the supernatant fraction (S). (F) Quantification of F/G-actin ratio in (E) (*n* = 6). Bars show mean ± SEM. Statistical significance was determined using the Mann-Whitney U test. Significant differences are indicated as ∗ for *p* < 0.05 and ∗∗∗ for *p* < 0.001.

We have previously demonstrated that nephrin signaling triggers localized actin polymerization,^20^^,^^21^^,^^28^ yet the effects on contractile forces are less characterized. To experimentally model and manipulate nephrin clustering at the SD (Figure 1A), we used a chimeric nephrin construct combining the extracellular region (ER) of CD16, the transmembrane region (TR) of CD7, and a GFP-tagged cytoplasmic region (CR) of nephrin (Figure 1B).^20^^,^^28^ The inclusion of this CD16 ER allows nephrin clustering and phosphorylation to be selectively induced through the addition of an anti-CD16 antibody (Figure 1B). This in turn can activate nephrin signaling in cultured podocytes which express very low levels of endogenous nephrin.^29^ Similar to previous reports,^20^^,^^28^ mouse podocyte cells (MPCs) expressing chimeric wild-type (WT) nephrin demonstrated increased nephrin YDxV tyrosine phosphorylation after 30 min of clustering, which could be detected by our anti-phosphonephrin Y1176/1193 and Y1217 antibodies (Figures 1C and 1D). As an indicator of actomyosin activation,^30^ we probed for phosphorylated myosin light chain 2 (pMLC2) at S19. In tandem with nephrin phosphorylation, we successfully observed an increased ratio of pMLC2 to total MLC2 (Figures 1C and 1D). Because actomyosin tension forces are associated with increased assembly and stability of filamentous actin (F-actin), as well as decreased severing into globular actin (G-actin),^31^^,^^32^ we then used an actin fractionation assay to detect the impacts of nephrin clustering on overall F-actin/G-actin ratios. In agreement with increased contractility, an increased proportion of F-actin to G-actin was observed following CD16 stimulation (Figures 1E and 1F). Together, these findings demonstrate that nephrin clustering and its phosphorylation at YDxV motifs elicits actomyosin activation and an overall increase in the proportion of F-actin to G-actin.

### Nephrin signaling induces robust force transmission that is dependent on its YDxV tyrosines

**Figure 2:**
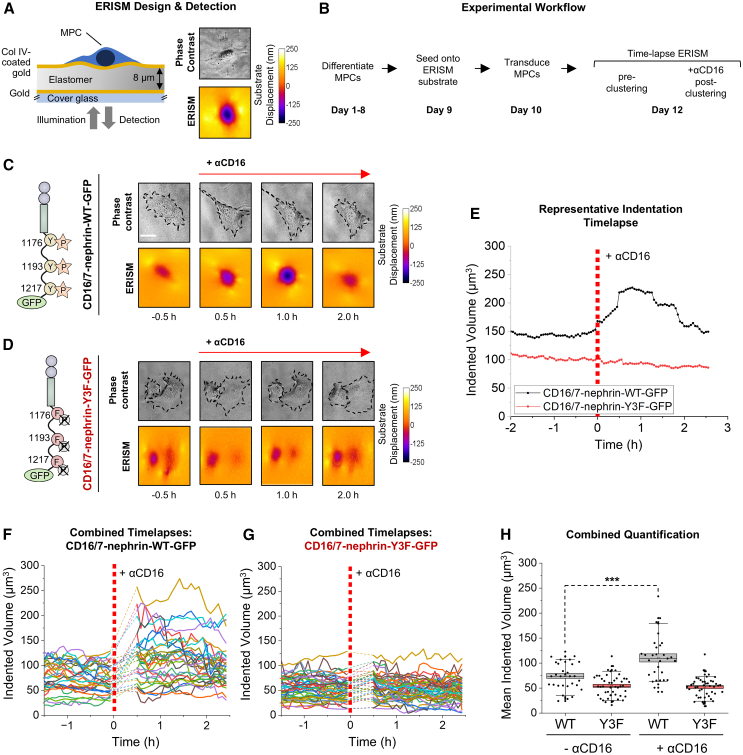
Nephrin phosphosignaling induces podocyte force transmission (A) Mouse podocyte cells (MPCs) were differentiated on Type IV collagen (Col-IV)-coated elastic resonator interference stress microscopy (ERISM) chips, made from an elastomer film sandwiched between two thin gold mirrors (gold thickness 10 and 15 nm, respectively). Forces exerted by the MPCs were imaged via the displacement they induced to the surface of the chip, while cell morphology was followed via simultaneous phase contrast imaging. The collagen coating on the ERISM chip was created with an apparent stiffness of 6.8 kPa, similar to the GBM. (B) Workflow for the visualization of MPC mechanotransduction using ERISM. (C) Representative ERISM timelapse images of a single CD16/7-nephrin-WT-transduced MPC showing increased force transmission after αCD16 stimulation, as measured by substrate displacement. (D) Representative ERISM timelapse images showing no change in force transmission after αCD16 stimulation in two MPCs transduced with CD16/7-nephrin-Y3F, in which the tyrosines (Y) at all 3 YDxV sites are mutated to phenylalanine (F) to prevent their phosphorylation. (E) Representative evolution of indented volume over time during clustering for an MPC transduced with CD16/7-nephrin-WT and an MPC transduced with CD16/7-nephrin-Y3F. (F) Change in indentation volume for multiple CD16/7-nephrin-WT-transduced MPCs following stimulation (*n* = 31 cells). (G) Change in indentation volume for multiple CD16/7-nephrin-Y3F-transduced MPCs following stimulation (*n* = 45 cells). (H) Analysis of time-averaged indentation volume for all analyzed MPCs (*n* = 31 for nephrin-WT, *n* = 45 for nephrin-Y3F) before and after stimulation. Box-and-whisker plots show mean (lines) ± SEM (boxes) 10–90% percentiles (whiskers). Statistical significance was determined using a paired Student’s *t* test. Significant differences are indicated as ∗∗∗ for *p* < 0.001. Scale bars in (C) and (D) are 50μm. See also Video S1 and Figure S1.

We next wanted to investigate the potential for nephrin signaling to transmit contractile forces in live intact cells. To this end, we employed elastic resonance interference stress microscopy (ERISM), which is a functional imaging modality that allows continuous monitoring of cell-substrate interactions and mapping of cellular forces. As previously validated,^33^^,^^34^ this method uses interference patterns forming in a gold-sandwiched elastomer film to detect and quantify force-induced displacement of a cellular substrate (see Figure 2A for a side view sketch of substrate displacement). To apply this technique here, we developed an MPC differentiation and viral transduction workflow to visualize force transmission effects of nephrin clustering (Figure 2B). As in prior studies,^34^ resting MPCs without anti-CD16 stimulation produced a substrate deformation pattern consisting of modest indentations underneath the cell body and expansions near the cell’s periphery (Figure 2C). This displacement pattern is characteristic of contractile forces that are transferred onto the substrate at the position of FAs, with the inward directed cell contraction leading to a slight compression of the elastomer substrate underneath the cell body.^33^^,^^34^ Upon addition of the anti-CD16 antibody, representative ERISM timelapse visualization showed that nephrin clustering was followed by a rapid and intense increase in substrate displacement along the same spatial pattern as resting cells, signifying a surge in overall force exertion (Figure 2C and Video S1).

Video S1. ERISM video capture of mouse podocyte cell (MPC) undergoing substrate displacement following CD16-nephrin-WT clustering, related to Figure 2C

To determine if these mechanical changes were dependent on nephrin’s YDxV tyrosines, we then used a previously designed CD16/7-nephrin-Y3F mutant with phenylalanine substituted at each of nephrin’s YDxV tyrosines (Figure 2D), preventing their phosphorylation and Nck recruitment.^28^ Pre-clustered MPCs transduced with the CD16/7-nephrin-Y3F mutant had a comparable force transmission pattern to CD16/7-nephrin-WT-transduced cells, but remarkably, did not elicit detectable changes in substrate displacement following anti-CD16 stimulation (Figure 2D).

The observations from the representative ERISM timelapse images and the relative effect between nephrin-WT and nephrin-Y3F were quantified by calculating the total volume of downward sensor deformation underneath the cell, the so-called indented volume (Figure 2E), which can be used as a proxy for the contractile force applied by the cell.^33^^,^^34^ The CD16/7-nephrin-WT-transduced MPCs showed a pronounced increase in force after addition of the anti-CD16 antibody with a broad peak 30–90 min after stimulus. By contrast, no significant increase in force is observed in MPCs transduced with the CD16/7-nephrin-Y3F mutant. This difference in peak substrate displacement can be observed with most timelapses that were used for quantification of cell behavior (Figures 2F and 2G), with 64.5% of CD16/7-nephrin-WT-expressing cells showing an increase in contractility of more than 20%, while only 15.6% of CD16/7-nephrin-Y3F-expressing cells showed this same reaction (Figure S1). Collectively, when comparing the time-averaged indented volume during the interval prior to anti-CD16 stimulation to the time-averaged indented volume post stimulus, we observed a significant increase in indented volume after clustering for nephrin-WT-transduced MPCs (*p* = 0.0004), but not for nephrin-Y3F-transduced MPCs (Figure 2H). To summarize, these findings demonstrate that nephrin relies on YDxV tyrosine phosphorylation to induce podocyte force transmission.

### Nephrin YDxV phosphorylation regulates focal adhesion dynamics

**Figure 3:**
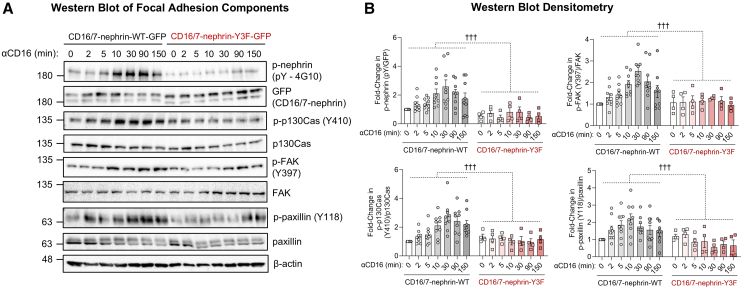
Nephrin YDxV tyrosine phosphorylation elicits adhesion signal activation (A) Western blot panel of nephrin and the adhesion proteins p130Cas, focal adhesion kinase (FAK), and paxillin at various time points following nephrin clustering in MPCs expressing nephrin-WT or nephrin-Y3F. Clustering of CD16/7-nephrin-WT by αCD16 stimulation led to significant activation of focal adhesion components, as measured by their increased phosphorylation. Peaks in activation can be observed following 30 min post-clustering. No activation is similarly observed when clustering CD16/7-nephrin-Y3F-transduced MPCs. (B) Densitometry calculations of phospho-proteins displayed in (A) (n = 4–9), normalized to β-actin. Bars show mean ± SEM. Statistical significance was determined using two-way ANOVA. Significant differences are indicated as ††† for *p* < 0.001.

FAs are key activators of contractility and actin organization,^35^ and using ERISM, previous studies have shown that the contractile forces in podocytes colocalize with the FA proteins vinculin and β1 integrin.^34^ Therefore, to assess whether nephrin can also alter FA signaling, we measured the phosphorylation of paxillin, along with the FA mechanosensors FAK and p130Cas in response to anti-CD16 stimulation. Phosphorylation of nephrin, paxillin, FAK and p130Cas were all increased in a time-dependent manner following induction of nephrin-WT, with peak activation occurring at 30 min (Figures 3A and 3B), reflecting the timing of peak force transmission observed via ERISM (Figure 2E). Also consist with ERISM data, the proportion of activated paxillin, FAK, and p130Cas slowly approached baseline after this peak, but most instances remained slightly elevated at the end of the 2.5 h timecourse (Figures 3A and 3B). Further, several of these signals began as early as 2 min post-clustering, highlighting the rapid nature of this relayed signal, and mirroring the onset of indentation on the ERISM timelapse (Figure 2E). In contrast, nephrin-Y3F clustering had no detectable adhesion signal activation after 2.5 h (Figures 3A and 3B). In support of this requirement for nephrin phosphosignaling, MPCs with double knockout of Nck1 and Nck2 (Nck1/2 DKO),^22^ both of which bind the YDxV motifs, showed suppressed phosphorylation of FAK and p130Cas upon CD16/7-nephrin-WT stimulation (Figure S2A), as well as nephrin, in line with the ability of Nck proteins to enhance nephrin phosphorylation.^36^ Intriguingly, transduction of either Nck1 or Nck2 could restore these responses following nephrin clustering, with a greater effect seen with Nck2 (Figure S2B). Thus, nephrin-induced force transmission appears to act simultaneously with the activation of central FA signaling modules, and this process is dependent on nephrin YDxV phosphorylation.

### Diabetic nephrin-Y3F mice develop exacerbated proteinuria

**Figure 4:**
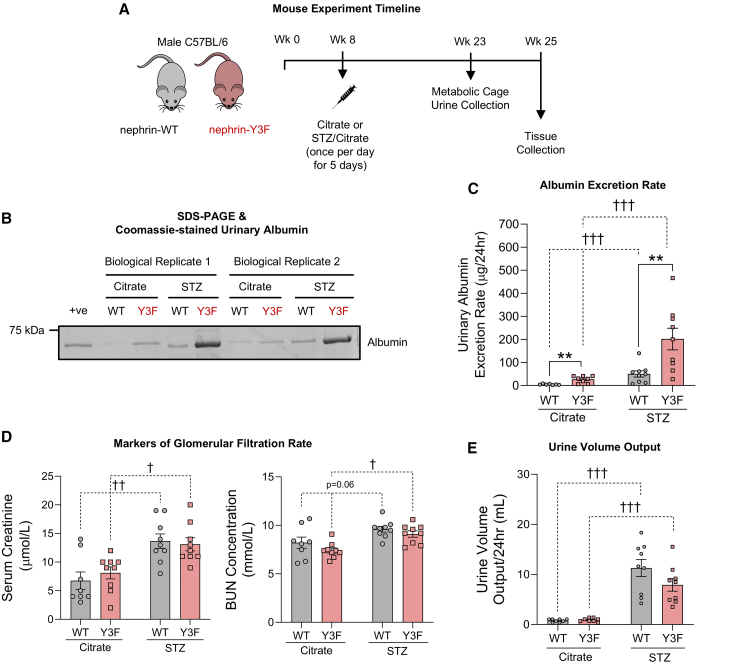
Nephrin-Y3F mice develop pronounced proteinuria upon diabetic injury (A) Experimental timeline for streptozotocin (STZ) diabetic challenge and tissue collection using nephrin-WT or nephrin-Y3F mice. (B) Coomassie-stained gel following SDS-PAGE of metabolic cage urine collections from citrate- or STZ-injected WT or nephrin-Y3F mice (two biological replicates shown). (C) Urinary albumin excretion rate calculated from metabolic cage urine collections shown in (B), showing greater proteinuria in diabetic nephrin-Y3F mice (n = 8–9 mice). (D) No differences in glomerular filtration rate were detected between WT and nephrin-Y3F genotypes, as estimated by serum creatinine and blood urea nitrogen (BUN; n = 8–9 mice). (E) 24-h urine volume output showed minimal differences between WT and nephrin-Y3F mice. Bars show mean ± SEM. Statistical significance was determined using two-way ANOVA with a *post hoc* Tukey’s test. Significant differences are indicated as † for *p* < 0.05, ∗∗/†† for *p* < 0.01, and ††† for *p* < 0.001.

Given the importance of YDxV tyrosines in modulating adhesion and force-generating signals *in vitro*, we next wanted to determine if these residues are utilized *in vivo* to mitigate glomerular pathologies associated with mechanical strain. We used the setting of type 1 diabetes (T1D) to model glomerular hyperfiltration, which results in increased shear and tension forces applied to podocytes.^37^^,^^38^ Previously characterized nephrin-Y3F knockin mice^39^ or wild-type (WT) controls were injected with low-dose streptozotocin (STZ) to induce T1D, with citrate buffer as a vehicle control (Figure 4A). Both WT and nephrin-Y3F mice had comparable diabetes induction, with similar HbA1c% and total units of insulin being required for treatment (Table S1). No differences were observed between genotypes for bodyweight and organ weights (kidney, heart, and liver), however all diabetic treatment groups had equivalent decreases in bodyweight and heart weight (Table S1). Assessment of urine collected from metabolic cages revealed that nephrin-Y3F mice had mildly elevated albuminuria compared to WT even in the absence of diabetic injury (Figures 4B and 4C), as previously observed.^39^ Further, diabetic WT mice had a level of albuminuria that was comparable to non-diabetic nephrin-Y3F mice. In contrast, diabetes induction in nephrin-Y3F mice led to a stark increase in albuminuria, with a much greater loss of protein compared to all other experimental groups observed (Figures 4B and 4C). Importantly, diabetic treatment groups had similar increases in serum creatinine and blood urea nitrogen (BUN) (Figure 4D), as well as equivalent 24-h urine volume output (Figure 4E), altogether suggesting comparable glomerular filtration rate and mechanical strain. Together, these findings demonstrate that DN worsens proteinuria in the absence of nephrin YDxV tyrosine phosphorylation.

### Glomerular sclerosis and hypertrophy, but not FP or GBM ultrastructure, are exacerbated by diabetes in nephrin-Y3F mice

**Figure 5:**
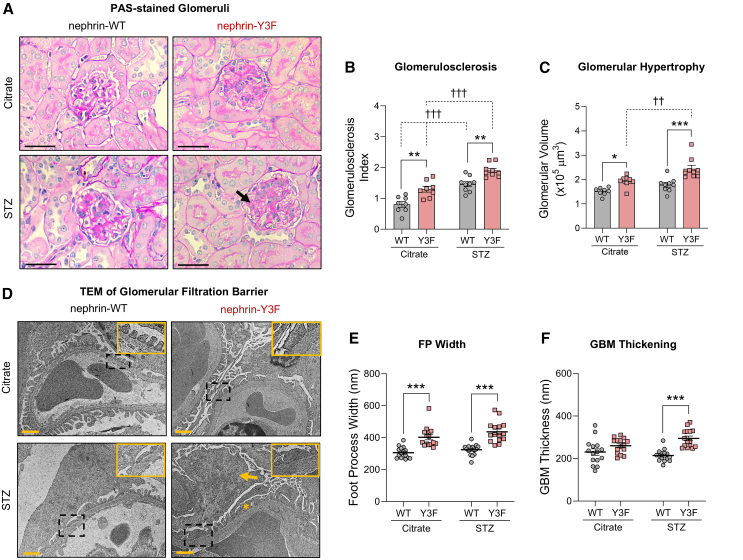
Diabetic nephrin-Y3F mice have increased glomerular sclerosis, increased hypertrophy, and ultrastructural changes to the filtration barrier (A) Representative periodic acid-Schiff (PAS)-stained glomeruli, with an arrow signifying the pronounced glomerulosclerosis in diabetic nephrin-Y3F mice. (B) Glomerulosclerosis index was calculated from PAS-stained sections shown in (A) using 20 cortical glomeruli per mouse (8–9 mice per group; n = 8–9). (C) Glomerular hypertrophy was estimated using glomerular volume of 20 cortical glomeruli per mouse (n = 8–9). (D) TEM images of the glomerular filtration barrier revealed moderate foot process effacement (asterisk) and podocyte hypertrophy (arrow) in non-diabetic and diabetic nephrin-Y3F mice. Foot process (FP) width (E) and glomerular basement membrane (GBM) thickness (F) were calculated from TEM images represented in (D). Scatterplots showing arithmetic means of all measurements from 15 peripheral capillary loop images, acquired across 3–5 glomeruli per mouse and 3 mice per group. Bars/lines show mean and error bars show SEM. Statistical significance was determined using two-way ANOVA with a *post hoc* Tukey’s test. Significant differences are indicated as ∗ for *p* < 0.05, ∗∗/†† for *p* < 0.01, and ∗∗∗/††† for *p* < 0.001. Scale bars in (A) and (D) are 40μm and 1μm, respectively.

To help explain this increase in proteinuria, we investigated glomerular structural changes using periodic acid-Schiff (PAS)-stained kidney sections. Diabetic nephrin-Y3F mice had significantly increased glomerulosclerosis scores and glomerular hypertrophy when compared with diabetic WT mice or citrate-treated Y3F controls (Figures 5A–5C). These phenotypes were primarily detected as expansion of the inner mesangium, consistent with lesions typically found in DN.^40^ Increased mesangial expansion and glomerulosclerosis scores were also observed in diabetic WT mice compared to citrate controls, although they only had slight increases in glomerular volume that did not reach significance (Figures 5A–5C), potentially explained by the known resistance to DN with the mouse C57BL/6 background.^41^ Non-diabetic nephrin-Y3F mice also presented a small degree of sclerosis and hypertrophy (Figures 5A–5C), in line with previous observations on this mouse background.^39^

Next, we examined the ultrastructure of the GFB to detect FP effacement and GBM matrix deposition. We first noted slightly larger podocyte projections and cell bodies in both non-diabetic and diabetic nephrin-Y3F mice, indicative of podocyte hypertrophy (arrow in Figure 5D). Concomitant with this hypertrophy, FP width and GBM thickness were significantly elevated in both non-diabetic and diabetic nephrin-Y3F mice. Unexpectedly, FP width and GBM thickness were only marginally greater in diabetic nephrin-Y3F mice, and were not significantly different when compared to non-diabetic nephrin-Y3F mice (*p* = 0.27 and *p* = 0.13, respectively) (Figures 5D–5F). However, we also did not observe increased FP effacement or GBM thickness in diabetic WT mice, possibly due to the aforementioned DN resistance on this genetic background (Figures 5D–5F). To summarize, nephrin-Y3F mice have greater FP effacement and GBM thickening, but these measures were not exacerbated in response to mechanical strain and the diabetic milieu, in contrast with their proteinuria, glomerulosclerosis, and glomerular hypertrophy.

### Diabetic nephrin-Y3F mice have decreased podocyte density, with increases in both podocyte size and podocyturia

**Figure 6:**
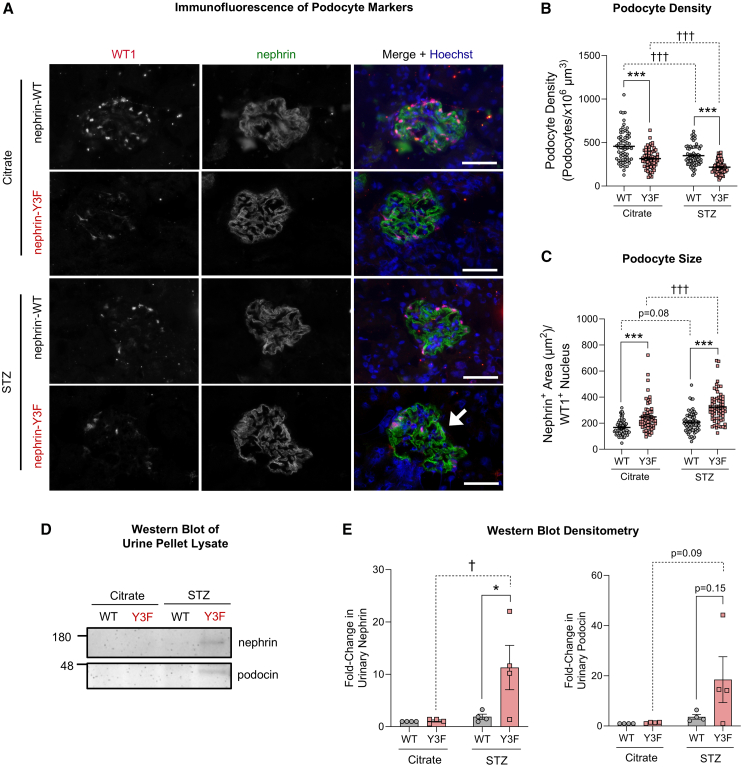
Diabetic nephrin-Y3F mice have decreased podocyte density, increased podocyte size, and prominent podocyturia (A) Representative immunofluorescence images of glomeruli stained with podocyte markers WT1 and nephrin in citrate- or STZ-injected WT or nephrin-Y3F mice. Merge images display pink podocyte nuclei when WT1 and Hoechst co-localize. Diabetic nephrin-Y3F mice show the greatest loss of podocytes and have notably larger distances along the glomerular periphery without detectable podocyte nuclei (arrow). (B) Podocyte nuclear densities calculated for a minimum of 20 glomeruli (10 cortical, 10 juxtaglomerular) per mouse with 3 mice used per group. (C) Podocyte size estimates for individual glomeruli, determined using nephrin area per WT1-stained nucleus. Data show a minimum of 20 glomeruli (10 cortical, 10 juxtaglomerular) per mouse with 3 mice used per group. (D) Representative western blot images of podocyte markers nephrin and podocin from metabolic cage urine pellet lysates (*n* = 4 mice). (E) Densitometric quantification of (C) showing podocytes in urine (podocyturia) of diabetic nephrin-Y3F mice. Bars show mean ± SEM. Statistical significance was determined using two-way ANOVA with a *post hoc* Tukey’s test. Significant differences are indicated as ∗/† for *p* < 0.05, and ∗∗∗/††† for *p* < 0.001. Scale bars in (A) are 40μm.

As YDxV tyrosine phosphorylation led to strong adhesion activation upon nephrin clustering (Figures 3A and 3B), we hypothesized that loss of nephrin phosphorylation would lead to podocyte detachment in nephrin-Y3F mice. To detect this potential loss of podocytes, we calculated podocyte density through the visualization of the podocyte markers nephrin and Wilms’ tumor 1 (WT1) in glomerular cross-sections. Reflecting nephrin’s impact on adhesion signaling, this visualization revealed a significant reduction in WT1+ nuclei per glomerular volume in diabetic nephrin-Y3F mice compared to diabetic WT and citrate-injected nephrin-Y3F mice (Figure 6A, with individual glomerular densities shown in Figure 6B). A significant reduction in podocyte densities was also observed in diabetic WT and citrate-injected nephrin-Y3F mice compared with citrate-injected WT mice (Figures 6A and 6B).

Importantly, diabetic nephrin-Y3F mice also presented with large areas along the GBM showing no apparent podocyte nuclei (arrow in Figure 6A), potentially indicative of compensatory podocyte hypertrophy and/or podocyte detachment. Therefore, we then measured this potential increase in podocyte size by calculating the nephrin-stained area per podocyte nucleus. Similar to the podocyte hypertrophy observed by TEM, nephrin-Y3F mice had increased podocyte size, with further increases being observed in diabetic conditions (Figure 6C). Next, reasoning that detached podocytes would be shed into the urine (podocyturia), we performed western blot analysis of cell-pelleted urine fractions using podocyte markers nephrin and podocin. As predicted, we observed a significant increase in the levels of urinary nephrin in diabetic nephrin-Y3F mice, along with a similar increase in podocin levels (Figures 6D and 6E). Little to no detection of nephrin or podocin was observed in all other experimental groups, including diabetic WTs and non-diabetic WT or nephrin-Y3F mice (Figures 6D and 6E). Taken together, loss of nephrin YDxV phosphorylation reduced podocyte density, increased podocyte hypertrophy, and led to podocyte detachment upon conditions of mechanical strain and diabetic injury.

## Discussion

Here, we have demonstrated the role of nephrin tyrosine phosphorylation in podocyte force transmission and activation of FA components. This signaling axis appears to play a role in disease contexts with increased shear and tension forces, and without this signaling ability during mechanical strain, kidneys are at increased risk of irreversible podocyte loss.

We first show that nephrin efficiently and spatiotemporally induces force transmission partly via the actomyosin network. Specifically, our detection of force transmission was concomitant with the phosphorylation of MLC2 (Figures 1C and 1D), suggesting the use of NM-IIa motors to generate tension.^30^ Loss of NM-IIa in podocytes is also associated with aberrant adhesion, contractility, and motility,^18^ which helps explain the cell detachment and proteinuria observed in diabetic nephrin-Y3F mice. Importantly, NM-IIa actomyosin fibers *in vivo* are absent in FPs, but present in primary processes and peri-nuclear areas.^17^ Since we cannot accurately map nephrin-NM-IIa signaling using the two-dimensional cell culture of ERISM, future studies should investigate this potential crosstalk between nephrin and contractility of major processes and peri-nuclear actomyosin fibers, as it could impact the cellular gene expression profile (e.g., via YAP nuclear translocation, which is downstream of nephrin signaling) and/or might serve to protect the nucleus from hydrodynamic shear forces generated by filtrate.^42^^,^^43^^,^^44^^,^^45^

Previously, clustered nephrin has been reported to use cortical actomyosin for lateral membrane movement,^27^ and we now demonstrate that nephrin signaling elicits broad contractile forces in podocytes. One possible mechanism explaining this effect is that nephrin YDxV phosphorylation might promote calcium-dependent contractility via PLCγ1.^46^ However, previous actomyosin induction from nephrin was dependent on Nck1/2 adaptors,^27^ which can signal to myriad mechanical effectors like dynamin, YAP, and various Rho GEFs/GAPs.^28^^,^^45^^,^^47^^,^^48^ Interestingly, loss of the Nck2 paralog alone leads to exacerbated podocyte injury, and Nck2 has unique binding partners implicated in contractility.^22^ For example, Nck2 can bind PLCε1,^49^ an activator of both calcium signaling and Rap1,^50^ and Rap1 has been previously implicated in nephrin-FA crosstalk via C3G and RapGEF2.^5^^,^^51^^,^^52^^,^^53^ Consistent with this notion, loss of both Nck adaptors prevented nephrin’s clustering-dependent activation of p130Cas and FAK, and re-expression of Nck adaptors, predominantly Nck2, led to recovery of this signaling axis from nephrin (Figure S2). Nevertheless, this recovery might have also been due to indirect functions of Nck adaptors, such as their enhancement of Fyn-mediated nephrin phosphorylation,^36^ which would have promoted an increase in all nephrin signals that drive contractility.

Next, nephrin can likely control peripheral forces via FA activation, as this peripheral force pattern has previously colocalized with the adhesion components vinculin and β1-integrin.^34^ We now show that nephrin clustering and YDxV phosphorylation alter the signaling dynamics of these FAs, as detected by the phosphorylation of paxillin, FAK, and p130Cas (Figure 3). These FA components are mechanosensitive and can allow FAs to mature via RhoA-mediated myosin II contractility, making them potent regulators of nascent adhesion maturation and leading edge migration.^13^^,^^54^^,^^55^ Therefore, it remains unclear whether nephrin independently elicits forces that stimulate FA mechanosensors, or if nephrin first signals the FAs (e.g., via Rap1) that in turn promote mechanical changes. In either case, synchronous changes occur in contractility from nephrin and FAs, as demonstrated by the similar timing between podocyte force transduction and adhesion signal activation (between Figures 2E, 2F, and 3). Notably, FAK, p130Cas, and other core FA proteins like α-actinin-4, Arp2/3, integrin-linked kinase (ILK), Nck, and Crk1/2 also localize to the SD,^20^^,^^56^^,^^57^^,^^58^ and future studies are needed to characterize how these cell-cell and cell-matrix adhesions can spatiotemporally regulate one another.

Furthermore, this study validates the importance of nephrin-stimulated force induction *in vivo*, as abolishing phosphorylation at YDxV motifs led to increased proteinuria and podocyte detachment in response to diabetic stress. Previously, nephrin YDxV phosphorylation was not necessary for the development of the SD but played an important role in injury from aging or experimental disease models.^39^^,^^59^^,^^60^ We now show that the YDxV signaling axis is utilized in mitigating diabetic nephropathy, which is the most common cause of chronic kidney disease and end-stage renal disease,^61^ and also an important model of mechanical strain. Highlighting the importance of mechanical strain in DN, glomerular hypertension and podocyte stretching in this pathology can be driven by renin-angiotensin system (RAS) activation,^37^^,^^38^^,^^62^ and decreasing these hemodynamic alterations via RAS or SGLT2 inhibitors has shown clinical efficacy.^63^

It has been suggested that podocytes partly counteract such mechanical changes by regulating a contractile buttressing force against the GBM, and loss of this force against the GBM can lead to excess protein leakage through this layer of the GFB.^63^^,^^64^ *In vivo*, this force could potentially arise from circumferential NM-IIa-containing major processes or actomyosin-containing sarcomeric structures in FP that arise during injury.^17^ Without proper contractile signaling, nephrin-Y3F mice likely have decreased control of this buttress force, resulting in mechanical strain-induced protein leakage through the GBM. This leakage could help explain why we observed marked proteinuria without exacerbated FP or GBM abnormalities in diabetic nephrin-Y3F mice (Figures 4B, 4C, and 5D–5F). Further, this loss of contractility could also help explain the extensive capillary loop dilation previously seen with nephrin-Y3F mice on outbred backgrounds,^39^ and their susceptibility to mesangial expansion (Figure 5A), as mesangial cell stretching can lead to increased matrix production.^40^ Interestingly, podocyte hypertrophy has also been linked to increased mechanical stretch,^65^^,^^66^ and the observed increase in size in nephrin-Y3F podocytes (Figures 5D and 6C) could be due to an inability to contract and counteract capillary tension forces.

**Figure 7:**
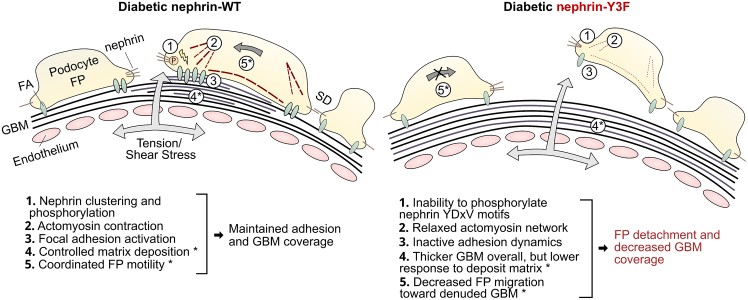
Illustration and summary of the podocyte response to mechanical stress in diabetic nephrin-WT and nephrin-Y3F mice Asterisk indicates that these functions are suggested by the present and previous studies but require further validation. Contractile fibers are shown in podocyte FP for simplicity, although regular contractile forces *in vivo* might be distributed along actomyosin-containing major processes. FP = Foot process; FA = focal adhesion; GBM = glomerular basement membrane; SD = Slit diaphragm.

Importantly, nephrin’s force induction is dependent on its clustering, which could allow for mechanosensation and detection of pathological glomerular alterations. While *trans*-interaction and clustering of nephrin’s extracellular regions might occur infrequently during normal glomerular function,^67^ it remains to be validated if clustering is engaged through pathological cues. Such cues include increased stress forces, altered regulation of nephrin interactors, or nephrin autoantibody circulation, which has been increasingly observed in patients with minimal change disease and idiopathic nephrotic syndrome.^68^^,^^69^ In the case of glomerular hypertension from DN, clustering-dependent YDxV phosphorylation elicits downstream signals that stimulate contractility, paxillin/FAK/p130Cas activation, nephrin and FA turnover, and migration.^28^^,^^49^^,^^70^^,^^71^ Collectively, these processes suggest subsequent FP expansion, and this expansion might operate as a transient adaptive change to counter stress forces and podocyte detachment, as previously hypothesized.^72^ Because our nephrin-Y3F mice did not have pronounced increases in FP width or GBM thickness under diabetic conditions (Figures 5D–5F), they might be less capable of adaptive FP expansion and matrix deposition in response to glomerular hypertension, allowing more areas for protein to leak through the GFB and less structural changes to prevent detachment. Although adaptive roles in FP expansion and matrix deposition remain to be validated, such roles are supported by the importance of actomyosin contractility in cell migration,^73^ and that nephrin has previously been shown to regulate exocytosis in an NM-IIa-dependent manner.^74^ Moreover, these roles could also suggest that remaining podocytes would then have a reduced ability to migrate to the detached areas, possibly explaining the wide areas without podocyte nuclei shown in Figure 6A. Taken together, these suggested mechanisms occurring downstream of nephrin phosphorylation that could be adaptive in DN are illustrated in Figure 7. Importantly, these functions are specific to DN and related pathologies, as citrate-injected nephrin-Y3F mice still develop moderate GBM thickening and effacement over time (Figures 5E and 5F), and charge-based injuries like protamine sulfate administration can lead to effacement in the absence of YDxV phosphorylation.^39^

Finally, alterations in this signaling axis are critical to the progression of DN and various podocytopathies. Notably, increased phosphatase activity (e.g., Shp1 and C1-Ten) leads to decreased nephrin phosphorylation and total levels in DN.^60^^,^^75^ However, these changes can be dependent on the stage of disease progression, with transient increases in nephrin expression and phosphorylation being observed in early stages of several glomerular diseases, followed by decreases in later stages.^69^^,^^76^^,^^77^ Thus, early injury might involve adaptive contractility and FA activation, but as the disease progresses decreases in nephrin phosphorylation would lead to exacerbated proteinuria and podocyte detachment. Interestingly, this progression in nephrin activity mirrors the alterations in glomerular filtration rate during DN,^37^ so further work should investigate how nephrin’s adaptive functions are linked to changes in mechanical strain. With this greater understanding of nephrin’s role in podocyte mechanobiology, newly revealed opportunities to clinically control podocyte mechanics and adhesion in many pathologies could be invaluable for renal health.

### Limitations of the study

Together, these results should be interpreted with consideration of their key limitations. One such limitation is the use of CD16 clustering and two-dimensional cell culture models. These models do not possess an SD, and structures like FAs largely differ from the podocyte microenvironment. Thus, it is possible that nephrin-dependent force transmission and FA crosstalk *in vivo* are less pronounced or require additional contextual cues. Furthermore, it remains unclear what stimuli leads to clustering *in vivo*, and other mechanisms of clustering might have different downstream effects than anti-CD16-mediated clustering *in vitro*. As another limitation, C57BL/6 mice have greater resistance against DN,^41^ and their absence of certain hallmarks (e.g., FP effacement and GBM thickening) make it challenging to discern if nephrin phosphorylation controls these processes during diabetic injury. Additionally, only a limited number of conditions were assessed (e.g., male mice with 17 week of STZ-mediated injury), and future studies could include female mice, shorter or longer durations of injury, and alternative models of mechanical strain, such as nephrectomy (e.g., UNx or 5/6 Nx) and/or deoxycorticosterone acetate (DOCA) salt-induced hypertension.^78^^,^^79^ Crucially, DN involves many factors that lead to injury (e.g., angiotensin-II, high glucose, TGF-β1),^38^ and these alternative injury models might help determine if the exacerbated injury in nephrin-Y3F mice was primarily related to mechanical strain or if these effects stem from the complex diabetic milieu. Lastly, we note that further assessment of podocyte detachment should be investigated, as podocyte density is only an indirect measure of detachment. Although diabetic nephrin-Y3F glomeruli had large regions along the GBM without WT1-stained nuclei, their decreased podocyte density also reflects their increased glomerular hypertrophy. More direct measures of podocyte detachment, such as detection of podocytes in spot urine, would be valuable to include in future studies.

## Resource availability

### Lead contact

Further information and requests for resources and reagents should be directed to and will be fulfilled by the lead contact, Nina Jones (jonesmcb@uoguelph.ca).

### Materials availability

• This study did not generate new unique reagents.
• All reagents, cell lines, and mouse strains are available upon request to the lead contact.

### Data and code availability

• All data reported in this paper will be shared by the lead contact upon request.
• This paper does not report original code.
• Any additional information required to reanalyze the data reported in this paper is available from the lead contact upon request.

## Acknowledgments

We kindly thank Dr. Elyse Roach and Dr. Ali Darbandi for their help with TEM sample preparation and imaging. We thank Jennifer Randall and additional staff at the University of Guelph Central Animal Facility for their help with mouse breeding, husbandry, and streptozotocin injections. Special thanks are also given to Dr. Jeremy Simpson and Dr. Jim Petrik (both University of Guelph) for their advisory role in experiment planning and data analysis. Lastly, we are grateful for the virus and antibody resources gifted by Dr. Tomoko Takano, Dr. Mira Krendel, and Dr. Louise Larose. This work was supported by the Canadian Institutes of Health Research (PJT-148731 to N.J.), 10.13039/501100000191Kidney Foundation of Canada (KFOC190002; to N.J.), the 10.13039/501100001804Canada Research Chairs Program (to N.J.), the 10.13039/501100000268Biotechnology and Biological Sciences Research Council (BB/P027148/1; to M.C.G and P.R.) and the Engineering and Physical Science Research Council (EP/P030017/1; to M.C.G.). Support to C.R.W. was provided by the National Sciences and Engineering Research Council of Canada Postgraduate Scholarship – Doctoral (NSERC PGS D) and a Graduate Entrance Excellence Scholarship from the 10.13039/100008986University of Guelph College of 10.13039/100000076Biological Sciences.

## Author contributions

C.R.W., C.E.M., P.L., L.A.N., N.M.K., M.C.G., P.A.R., and N.J. conceived and designed experiments. C.R.W. performed experiments in Figures 4, 5, and 6 and Table S1. C.E.M. performed experiments in Figures 1, 3, and S2. J.D.K. performed experiments in Figures 2 and S1. L.A.N. and P.L. provided assistance with experiments for Figure 4 and Table S1. C.E.M., N.M.K., M.C.G., and P.A.R. provided assistance with experiments for Figures 2 and S2. C.E.M. performed data analysis in Figures 1 and 3. J.D.K. and N.M.K. performed data analysis in Figures 2 and S1. C.R.W. performed data analysis in Figures 4, 5, and 6 and Table S1. A.Y.W. performed data analysis in Figure 5 and Table S1. C.R.W. assembled all tables and figures, with assistance from C.E.M for Figures 1, 2, and 3, and J.D.K and N.M.K. for Figure 2. C.R.W. wrote the manuscript with N.J. and all authors approved the final version.

## Declaration of interests

N.M.K. and M.C.G. are the inventors of a patent on the ERISM technology.

## STAR★Methods

### Key resources table

**Table:** 

REAGENT or RESOURCE	SOURCE	IDENTIFIER
**Antibodies**		

Mouse anti-phosphotyrosine (clone 4G10)	Upstate Biotechnology	Cat #: 16-101; RRID: AB_310776
Mouse anti-green fluorescent protein (clone B2)	Santa Cruz Biotechnology	Cat #: sc-9996; RRID: AB_627695
Rabbit anti-phosphoS19 myosin light chain 2	Cell Signaling Technology	Cat #: 3671; RRID: AB_330248
Rabbit anti-MLC2	Cell Signaling Technology	Cat #: 3672; RRID: AB_10692513
Rabbit anti-phosphoY410 p130Cas	Cell Signaling Technology	Cat #: 4011; RRID: AB_2274823
Mouse anti-p130Cas	BD Biosciences	Cat #: 610272; RRID: AB_397667
Rabbit anti-phosphoY397 focal adhesion kinase	Cell Signaling Technology	Cat #: 3283; RRID: AB_2173659
Rabbit anti-FAK (clone EP695Y)	Sigma-Aldrich	Cat #: 04-591; RRID: AB_838267
Rabbit anti-phosphoY118 paxillin	Cell Signaling Technology	Cat #: 2541; RRID: AB_2174466
Rabbit anti-paxillin (clone Y113)	Abcam	Cat #: ab32084; RRID: AB_779033
Mouse anti-CD16 (clone 3G8)	Santa Cruz Biotechnology	Cat #: sc-19620; RRID: AB_626924
Rabbit anti-podocin	Sigma-Aldrich	Cat #: PO372; RRID: AB_261982
Mouse anti-β-actin (clone AC15)	Sigma-Aldrich	Cat #: A1978; RRID: AB_476692
HRP-linked goat anti-rabbit	Bio-Rad	Cat #: 170-6515; RRID: AB_11125142
HRP-linked goat anti-mouse	Bio-Rad	Cat #: 170-6516; RRID: AB_11125547
Rabbit anti-phosphoY1176/Y1193 nephrin	Abcam	Cat #: ab80299; RRID: AB_1580757
Rabbit anti-phosphoY1217 nephrin (clone EPTPG3)	Abcam	Cat #: ab80298; RRID: AB_1603375
Rabbit anti-nephrin	Li et al., 2004^80^	N/A
Rabbit anti-Nck (1793)	Lussier and Larose 1997^81^	N/A
Guinea pig anti-nephrin	Fitzgerald Industries International	Cat #: 2OR-NP002; RRID: AB_1288100
Rabbit anti-Wilms Tumor 1 (C-19)	Santa Cruz Biotechnology	Cat #: sc-192; RRID: AB_632611
Goat anti-guinea pig (Alexa Fluor 488)	ThermoFisher Scientific	Cat #: A11073; RRID: AB_2534117
Donkey anti-rabbit (Alexa Fluor 647)	ThermoFisher Scientific	Cat #: A31573; RRID: AB_2536183

**Bacterial and virus strains**		

AdEasy XL adenoviral system	Agilent Technologies	Cat #: 240010
CD16/7-nephrin-WT-GFP adenovirus	Martin et al., 2020^28^	N/A
CD16/7-nephrin-Y3F-GFP adenovirus	Martin et al., 2020^28^	N/A
hNCK1-flag adenovirus	Martin et al., 2022^22^	N/A
hNCK2-flag adenovirus	Martin et al., 2022^22^	N/A
Flag control adenovirus	Martin et al., 2022^22^	N/A

**Chemicals, peptides, and recombinant proteins**		

Recombinant mouse interferon gamma (IFN-γ)	PeproTech	Cat #: 315-05
Cultrex rat collagen I	R&D Systems	Cat #: 3440-100-01
Cultrex mouse collagen IV	R&D Systems	Cat #: 3410-010-01
Teklad standard chow diet (14% fat)	Inotiv	Cat #: 2914
Teklad 18% fat chow diet	Inotiv	Cat #: 2918
Streptozotocin (STZ)	Sigma-Aldrich	Cat #: S0130
ProZinc protamine zinc recombinant human insulin	Boehringer Ingelheim	DIN #: 02405202
Mouse serum albumin (MSA) standard	ExoCell	Cat #: 1011

**Critical commercial assays**		

G-Actin/F-Actin *In Vivo* Assay kit	Cytoskeleton, Inc.	Cat #: BK037
Mouse HbA1c kit	Crystal Chem	Cat #: 80310
Cobas UREAL Assay	Roche	Cat #: 04460715
Creatinine Assay	Randox Laboratories Ltd.	Cat #: CR2336

**Experimental models: Cell lines**		

Immortalized mouse podocyte cells (MPCs)	Martin et al., 2020^28^	N/A
Nck1 and Nck2 double KO (DKO) mouse podocyte cells (MPC)	Martin et al., 2022^22^	N/A

**Experimental models: Organisms/strains**		

C57BL/6NCrl-Elite-SOPF mice	Charles River	Strain code: 475; RRID: IMSR_CRL:475
C57BL/6N *Nphs1*^Y3F/Y3F^ mice	New et al., 2016^39^	MGI: 7624348

**Oligonucleotides**		

*Nphs1*^Y3F^-Fw (genotyping) 5′-GCATATGTGAACGCATGAGG-3′	New et al., 2016^39^	N/A
*Nphs1*^Y3F^-Rv (genotyping) 5′-GAAGGTGGTTGGTTGCAGTT -3′	New et al., 2016^39^	N/A

**Software and algorithms**		

ToupView	ToupTek Photonics	RRID: SCR_017998
GraphPad Prism Version 10.0.2	GraphPad	RRID: SCR_002798
ImageJ	National Institutes of Health	RRID: SCR_003070
Inkscape	N/A	RRID: SCR_014479

**Other**		

Cobas c501 analyzer	Roche	RRID: SCR_025255
Leica DM1000	Leica	RRID: SCR_020225
FEI Tecnai G2 F20	FEI	RRID: SCR_021365
Echo Revolve	BICO	Model: RVL2-K2

### Experimental model and study participant details

#### Cell lines and culture conditions

Mouse podocyte cells (MPCs), along with Nck DKO MPCs, were generated from the Immortomouse (Charles River, designated CBA;B10-Tg[H2Kb-tsA58]6Kio/Crl) as previously described.^22^^,^^82^ MPCs were grown at 33°C and 5% CO_2_, while cultured in Dulbecco’s modified Eagle’s medium/F12 (DMEM/F12) (Hyclone), 10% fetal bovine serum (FBS, Hyclone), 10 U/mL recombinant mouse interferon-γ (IFN-γ, Peprotech), 200 U/mL penicillin, and 20 μg/mL streptomycin (Invitrogen). For MPC differentiation, cells were seeded on type-I collagen (R&D Systems) and placed at 37°C in media containing 2% FBS media (without IFN-γ) for 10-14 days. Cells were regularly tested for *Mycoplasma spp.* and found to be negative.

#### Animal care and study approval

Animal housing protocols were maintained in accordance with the guidelines set by the Canadian Council of Animal Care. Approval for all experimental procedures involving mice was provided by the Animal Care Committee at the University of Guelph (AUP 4181). Prior to experimentation, mice were housed with a 12-hour light/12-hour dark cycle and given free access to standard chow diet (Teklad 2914, Inotiv) and drinking water.

C57BL/6 nephrin-Y3F knockin mice were previously generated and validated as previously described.^39^ Nephrin-Y3F mice carried three Y→F missense mutations (p.Y1191F, p.Y1208F, and p.Y1232F), which correspond to the human tyrosine residues Y1176, Y1193, and Y1217, respectively. To refresh the colony genetic background, nephrin-Y3F mice were backcrossed with WT C57BL/6NCrl-Elite-specific and opportunistic pathogen free (SOPF) mice (Charles River) every 5-10 generations. Mice were genotyped before experimentation by polymerase chain reaction (PCR) and resolved on 2% agarose gels.^39^ Littermates (8-10 weeks of age) were randomly selected for each treatment group, and a minimum of 2 litter groups were used for all experimental comparisons. Only male mice were used due to low susceptibility to streptozotocin in female mice.^83^

### Method details

#### Transductions and clustering stimulation

Constructs containing CD16/7-nephrin-WT-GFP, CD16/7-nephrin-Y3F-GFP, NCK1-flag, or NCK2-flag were assembled using the human sequences of nephrin or NCK and validated as previously described.^20^^,^^84^ Adenoviruses containing these constructs (or flag control) were generated using the AdEasy XL Adenovirus system (Agilent Technologies) and transduced into differentiated MPCs.^84^ Viruses were transduced for 16 hours at a 1:100 dilution in DMEM/F12 with 2% FBS. Transduction media was replaced with 2% FBS-containing DMEM/F12 for 15 hours prior to CD16-clustering stimulation. Cells were then incubated with 200 ng/mL of anti-CD16 antibody (clone 3G8, sc19620, Santa Cruz Biotechnology) in 10% FBS-containing DMEM/F12 for 30 minutes and then processed for visualization or cell lysis.

#### Elastic Resonator Interference Stress Microscopy

MPCs were visualized using Elastic Resonator Interference Stress Microscopy (ERISM) as previously described.^34^ On day 9 of MPC differentiation, cells were seeded on a type-IV collagen-coated ERISM chip at a density of 500 cells per well. Cells were infected with nephrin fusion protein-containing adenovirus on day 10 of differentiation, with media replaced on day 11, and time-lapse force measurement, along with CD16 clustering stimulation, was carried out over 4 hours on day 12. Phase-contrast images were used to define cell margins and indentation volumes were calculated for singular cells as described in prior studies.^33^ GFP fluorescence was confirmed for each cell being quantified. The primary data provided by ERISM is a deformation map, i.e. spatially resolved information on how much cells deform the compliant ERISM chip on which they are cultured. To infer the overall contractile force exerted by individual cells from the deformation map, the total indented volume per cell was calculated by integrating the chip deformation across the entire area of downward/negative indentation. A drift correction of the time-lapse data was applied using the the Fiji macro Fast4DReg.^85^

#### Cell lysis and protein extraction

MPCs were placed on ice and lysed in PLC+ lysis buffer (50 mM N-2-hydroxyethylpiperazine-N′-2-ethanesulfonic acid [HEPES], 10% glycerol, 1 mM ethylene glycol tetraacetic acid (EGTA), 10 mM NaPPi, 1.5 mM MgCl_2_, 150 mM NaCl, 100 mM NaF, and 1% Triton X-100) containing protease inhibitors (0.1 mg/mL aprotinin and 0.1 mg/mL leupeptin) and 1 mM sodium orthovanadate and 1 mM phenylmethanesulfonyl fluoride (PMSF). Cell lysates were then sonicated using a Vibra-Cell (Sonics & Materials Inc.) for 5-10 seconds, incubated on ice for 5 minutes, and centrifuged at 14,000 x g for 10 minutes at 4°C. Supernatants were collected and protein concentrations were quantified using a bicinchoninic acid (BCA) protein assay (Pierce). Samples were then mixed with appropriate volumes of 5X sodium dodecyl sulphate (SDS) sample buffer (50% glycerol, 300 mM tris(hydroxymethyl)aminomethane [Tris], 10% SDS, and 25% β-mercaptoethanol [BME], 0.3 mg/mL bromophenol blue, pH 6.8) and denatured at 100°C for 5 minutes.

#### Immunoblotting

Whole cell lysates (10-50 μg) were loaded and resolved on 8-12% SDS-polyacrylamide gels (SDS-PAGE) and transferred to polyvinylidene (PVDF) membranes (EMD Millipore) for 1 hour at 100 volts. Membranes were blocked with 5% bovine serum albumin (BSA, Roche) or 5% non-fat milk powder in Tris-buffered saline with Tween (TBST, with 0.15 M NaCl, 0.02 M Tris, and 0.005% Tween-20 [Sigma-Aldrich]) for 1 hour at room temperature or 1-2 days at 4°C. Blocked membranes were then incubated with primary antibody for 1 hour at room temperature or overnight at 4°C with rocking. Membranes were then washed and incubated with horseradish peroxidase (HRP)-linked secondary antibody in TBST for 1 hour at room temperature. Membranes were washed again and signals were detected using enhanced chemiluminescence (ECL, Pierce) or Luminata Crescendo (EMD Millipore) and imaged on a ChemiDoc XRS+ (Bio-Rad). Densitometry was performed using *ImageLab* software (Bio-Rad).

The following commercial antibodies were used for immunoblotting: mouse anti-phosphotyrosine 4G10 (16-101, 1:1000, Upstate Biotechnology), mouse anti-green fluorescent protein B2 (GFP, sc-9996, 1:1000, Santa Cruz Biotechnology), rabbit anti-phosphoS19 myosin light chain 2 (MLC2, 3671, 1:1000, Cell Signaling Technology), rabbit anti-MLC2 (3672, 1:1000, Cell Signaling Technology), rabbit anti-phosphoY410 p130Cas (4011, 1:1000, Cell Signaling Technology), mouse anti-p130Cas (610272, 1:1000, BD Biosciences), rabbit anti-phosphoY397 focal adhesion kinase (FAK, 3283, 1:1000, Cell Signaling Technology), rabbit anti-FAK (04-591, 1:1000, Cell Signaling Technology), rabbit anti-phosphoY118 paxillin (2541, 1:1000, Cell Signaling Technology), rabbit anti-paxillin (Y113, ab32084, 1:1000, Abcam), rabbit anti-podocin (PO372, 1:1000, Sigma-Aldrich), mouse anti-β-actin (AC15, A1978, 1:5000, Sigma-Aldrich), HRP-linked goat anti-rabbit (170-6515, 1:7500, Bio-Rad), and HRP-linked goat anti-mouse (170-6516, 1:10,000, Bio-Rad). All specific nephrin, phosphonephrin, and Nck (1793) antibodies were used at 1:1000 and were generated and validated previously.^80^^,^^81^^,^^86^

#### Actin sedimentation assay

Filamentous-actin (F-actin) to globular-actin (G-actin) ratios were measured using the G-Actin/F-Actin *In Vivo* Assay kit (BK037, Cytoskeleton, Inc.) as per the manufacturer’s instructions. Differentiated MPCs were placed in F-actin stabilization buffer, homogenized, and centrifuged. Pellet fractions (containing F-actin) and supernatant fractions (containing G-actin) were loaded on SDS-PAGE gels and visualized via western blot (as detailed above).

#### Streptozotocin injury model

For diabetes induction, we used a chronic model of type 1 diabetes using a low-dose streptozotocin (STZ) regimen, as previously detailed.^83^ At 8-10 weeks-of-age, male mice were fasted for 4-5 hours and intraperitoneally administered citrate buffer (0.05 M sodium citrate, pH 4.5, 0.2 μm-filter-sterilized) or STZ (Sigma-Aldrich) in citrate buffer at a dose of 55 mg/kg, at one dose per day for five days. A 7 mg/mL STZ solution was prepared in citrate buffer no more than 5-15 minutes before injection. Mice were given 1% sucrose in drinking water for 20 hours following their first injection to prevent hypoglycemic shock. One week prior to STZ administration, all mice were switched to 18% fat chow (Teklad 2918, Inotiv). Blood glucose levels were regularly assessed by tail needle-prick and mice were administered insulin subcutaneously to maintain blood glucose below 27 mM. Mice were sacrificed at 25-weeks-of-age by CO_2_ asphyxiation.

#### Whole blood and serum assessment

Immediately following mouse sacrifice, blood was collected by cardiac puncture and placed in collection tubes containing EDTA (41.1395.105, Sarstedt) or clotting gel (41.1378.005, Sarstedt). Whole blood was placed at 4°C for no longer than one week and hemoglobin A1C (HbA1c) percentage was quantified using the Mouse HbA1c Kit by Crystal Chem (80310) as per manufacturer’s instructions. Clotting tubes were placed at room temperature for 3 hours and centrifuged at 400 x g for 10 minutes. Supernatants were collected and snap-frozen on dry ice for storage before quantification. Serum was analyzed at the Animal Health Laboratory – Clinical Pathology at the University of Guelph. Colorimetric assay of blood urea nitrogen (BUN) and serum creatinine was performed using the Cobas UREAL Assay (04460715) and Randox Creatinine Assay (CR2336) respectively on a Cobas c501 Analyzer (Roche).

#### Evaluation of proteinuria

At 23-weeks-of-age, mice were placed in metabolic cages (Tecniplast) for 17 hours overnight and urine volumes were recorded. Urine samples were centrifuged at 2500 x g for 20 minutes, supernatant and pellet fractions were separated, snap-frozen on dry ice, and stored at -20°C. Protein concentration was performed using 200 μL of urine by methanol-chloroform precipitation.^87^ Air-dried pellets were resuspended in 20 μL diH_2_O, mixed with equivalent volume of 2X SDS sample buffer, and denatured for 5 min at 100°C. 20 μL of concentrated protein sample was loaded on 10% SDS-PAGE gels, and 0.5 μg of mouse serum albumin (MSA, ExoCell) was loaded as a positive control. Gels were stained using Coomassie Brilliant Blue (Sigma-Aldrich) for 45 minutes, washed twice with destaining solution (45% methanol and 10% glacial acetic acid) for 10 minutes at room temperature, before washing with destaining solution overnight. Densitometry was performed using *ImageJ* software and urinary albumin was normalized to the MSA standard.

#### Histology

Coronal kidney sections were fixed in 10% formalin for 48 hours and submitted to the Animal Health Laboratory – Histotechnology for processing. Kidneys were paraffin-embedded and 2 μm sections were stained using periodic acid-Schiff stain to visualize glomerular matrix and basement membranes. A minimum of 20 cortical glomeruli per mouse were visualized on a Leica DM1000 using the *ToupView* software (ToupTek). Glomerular volume was estimated by the method established by Weibel and Gomez, assuming a shape constant of 1.38 for a sphere, and a 10% coefficient of variation.^88^^,^^89^ Glomerulosclerosis index was quantified using a previously established weighted-scoring system dependent on the percentage of sclerosed area per glomerulus.^90^

#### Transmission electron microscopy

Kidney cortices were dissected into pieces approximately 1 mm^3^ in volume and placed in 0.1 M sodium cacodylate fixative containing 2% glutaraldehyde and 4% paraformaldehyde (Electron Microscopy Sciences). After a minimum of 48 hours, samples were transferred into 0.1 M sodium cacodylate buffer for storage and submitted to SickKids Nanoscale Biomedical Imaging Facility for processing. Samples were post-fixed with 1% osmium tetroxide, dehydrated, and embedded in Quetol-Spurr resin. 90 μm ultrathin sections were stained with uranyl acetate and lead citrate and visualized on a FEI Tecnai G2 F20 at the University of Guelph Molecular and Cellular Imaging Facility. For each mouse, a minimum of 15 peripheral capillary loops across 3-5 glomeruli were imaged and used for subsequent analysis. Ultrastructural measurements were performed using *ImageJ* software. GBM thickness was quantified using the orthogonal intercept method with a grid area of 2.25 μm^2^,^91^ and FP width was quantified by measuring the distance between basolateral edges of FPs localized to peripheral capillary loops.

#### Podocyte density, size, and podocyturia

For immunofluorescence (IF) imaging, coronal kidney sections were placed in Shandon Cryomatrix (Richard-Allan Scientific), snap-frozen on dry-ice, and stored at -80^o^C. 6 μm sections were fixed in cold acetone for 10 minutes, washed with PBS (137 mM NaCl, 2.7 mM KCl, 10 mM Na_2_HPO_4_, and 1.8 mM KH_2_PO_4_, pH 7.4), and blocked with 10% normal goat serum (NGS, Santa Cruz Biotechnology) in PBS for 1 hour at room temperature. Sections were incubated with primary antibodies in 10% NGS for 1 hour at room temperature using guinea pig anti-nephrin (2OR-NP002, 1:200 Fitzgerald) and Wilms Tumor 1 (WT1; C-19, sc-192, 1:50, Santa Cruz Biotechnology), with PBS washing before and after all steps. Sections were light-protected and incubated with secondary antibodies in 10% NGS for 45 minutes at room temperature using goat anti-guinea pig (Alexa Fluor 488, A11073, 1:400, ThermoFisher Scientific), and donkey anti-rabbit (Alexa Fluor 647, A31573, 1:400, ThermoFisher Scientific). Sections were stained with Hoechst 33258 (H1398, 1:2000, Invitrogen) for 2 minutes at room temperature to stain nuclei and mounted using ProLong Diamond Antifade Mountant (Invitrogen). For each mouse, images of 10 cortical and 10 juxtamedullary glomeruli were taken on an Echo Revolve (Model RVL2-K2) and analyzed using *ImageJ* software. Podocyte density was quantified using apparent mean nuclear caliper diameter and WT1+ nuclear counts, as previously described.^92^ To measure podocyte size, nephrin-stained images underwent background reduction, thresholding, and the wand tool was used to select nephrin-stained regions and subtract internal unstained areas. This area was then normalized to the podocyte count of the selected glomerulus.

To detect podocyturia, snap-frozen pellets from metabolic cage urine collection (detailed above; following 2500 x g for 20 minutes at room temperature) were resuspended in 200 μL of PLC+ lysis buffer, sonicated using a Vibra-Cell (Sonics & Materials Inc.) for 5 seconds, and centrifuged at 12,000 x g for 10 minutes. Samples were mixed with SDS sample buffer and denatured at 100°C for 5 minutes. 35 μL of pellet lysate was run on 10% SDS-PAGE gels, transferred onto PVDF membranes, and immunoblotted as described above.

### Quantification and statistical analysis

All figures and statistical analyses were performed using *GraphPad Prism* software (version 9). Values are presented as mean ± SEM. Statistical significance before and after CD16 clustering was calculated using a paired Student’s *t*-test. All other significant differences were determined using a Mann-Whitney U test when between two non-parametric groups or a two-way ANOVA with a *post hoc* Tukey’s Honest Significant Difference (HSD) test where applicable. Significance was established using an α level of 0.05 and visualization of significant differences for separate factors were distinguished using asterisks (∗) for comparisons within individual treatment groups or obelisks (†) for comparisons within genotypes.
