# Supplementary material for: Reduced nephrin tyrosine phosphorylation impairs podocyte force transmission and accelerates detachment in disease
Source: iScience. 2025 May 14;28(6):112673. doi: 10.1016/j.isci.2025.112673 (PMC12159891; doi:10.1016/j.isci.2025.112673)
Supplement: Document S1. Figures S1 and S2 and Table S1 [file mmc1.pdf]

iScience, Volume *28*

Supplemental information

Reduced nephrin tyrosine phosphorylation impairs podocyte force transmission and accelerates detachment in disease

Casey R. Williamson, Claire E. Martin, J. Dinesh Kumar, Peihua Lu, Laura A. New, Alice Y. Wang, Nils M. Kronenberg, Malte C. Gather, Paul A. Reynolds, and Nina Jones

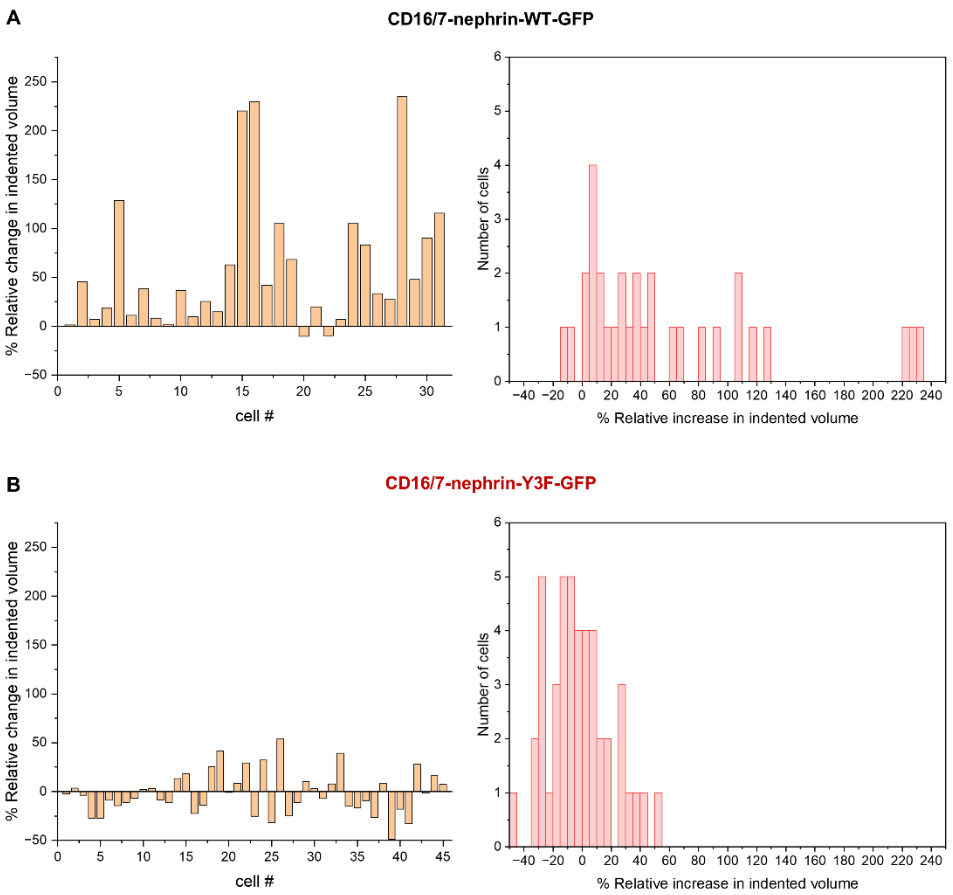

Figure S1. Relative change in indented volumes measured by ERISM for individual cells before and after CD16/7-nephrin clustering (A) The percent change in indented volume before and after anti-CD16 addition in CD16/7-nephrin-WT-GFP-transduced mouse podocyte cells (MPCs). This figure corresponds to the data collected in Figure 2F. Left graph shows the relative change in sequential order for each individual cell, while the right graph is a histogram of the number of cells in each range of percent relative change. **(B)** The percent change in indented volume before and after anti-CD16 addition in CD16/7-nephrin-Y3F-GFP-transduced MPCs. This figure corresponds to the data collected in Figure 2G. Left graph shows the relative change in sequential order for each individual cell, while the right graph is a histogram of the number of cells in each range of percent relative change.

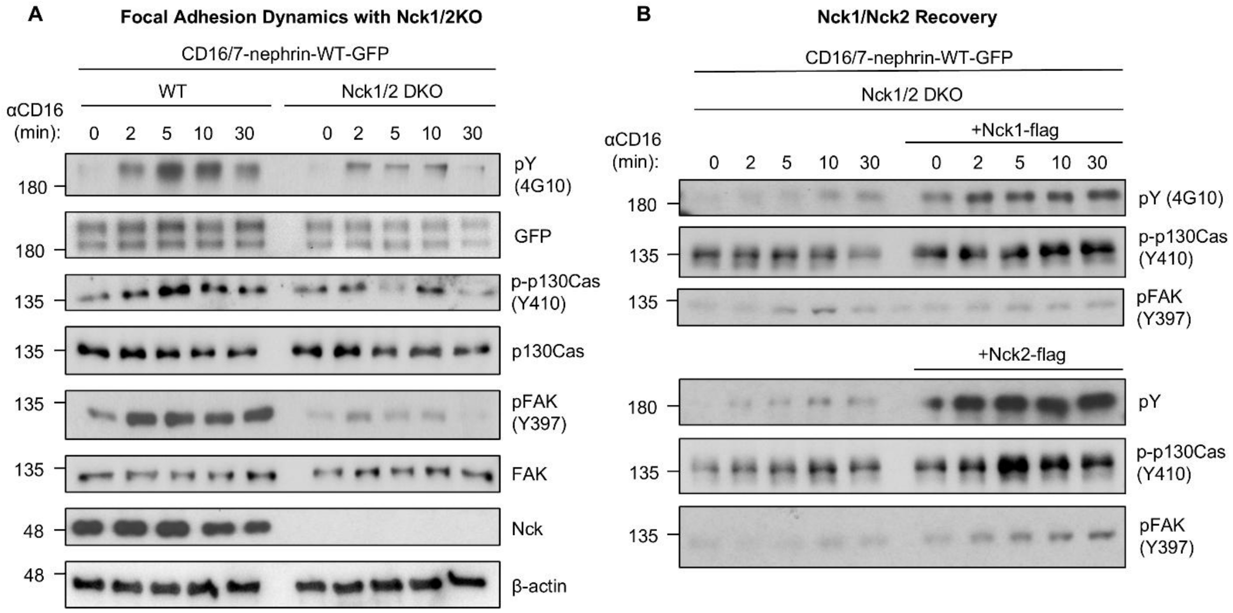

Figure S2. Nephrin-dependent adhesion activation is modulated by Nck2 expression. (A) Western blot analysis of phosphorylated and total p130Cas and focal adhesion kinase (FAK), before and after nephrin clustering in wild-type (WT) and Nck1/2 double knockout (DKO) mouse podocyte cells (MPCs). Following nephrin clustering via anti-CD16 antibody (αCD16), phosphorylated p130Cas and FAK are increased in WT MPCs, with only minimal or no increases in Nck1/2 DKO MPCs (n=1-2). **(B)** Phosphorylated p130Cas and FAK were assessed via western blot after nephrin clustering in Nck1/2 DKO MPCs, with or without re-expression of the single Nck paralogs, Nck1 or Nck2 (n=1-2). In DKO MPCs transduced with Nck1-expressing adenovirus, no recovery of p130Cas or FAK can be observed upon nephrin clustering. In contrast, DKO MPCs transduced with Nck2-expressing adenovirus showed full recovery of nephrin clustering-dependent p130Cas activity, with partial recovery of FAK activity as well.

Table S1. Weight and physiological characteristics of nephrin-Y3F mice following streptozotocin (STZ)-induced diabetes

	WT-Citrate	Y3F-Citrate	WT-STZ	Y3F-STZ
Bodyweight AUC	3328.69 ± 115.94[a]	3212.17 ±153.11[a]	2860.77 ± 85.67[b]	2831.14 ± 101.99[b]
HbA1c (%)	4.28 ± 0.11[A]	4.07 ± 0.14[A]	9.92 ± 0.30[B]	9.18 ± 0.35[B]
Total Lifetime Insulin Administered (U)	N/A	N/A	50.0 ± 2.7	47.9 ± 3.6
KW/TBL (g/cm)	0.227 ± 0.007	0.253 ± 0.007	0.246 ± 0.013	0.259 ± 0.017
HW/TBL (g/cm)	0.083 ± 0.004[a]	0.089 ± 0.003[a]	0.071 ± 0.003[b]	0.074 ± 0.004[b]
LW/TBL (g/cm)	0.878 ± 0.027	0.981 ± 0.035	0.965 ± 0.033	0.988 ± 0.040

Data is shown using mean ± SEM (n=8-9). Statistical significance was determined using two-way ANOVA with a *post hoc* Tukey's test. Separate letters note significant differences, where lowercase letters indicate $p < 0.05$, and uppercase letters indicate $p < 0.001$. AUC = area under the curve; KW = kidney weight; TBL = tibia length; HW = heart weight; LW = liver weight
